# An Interactive Pharmacokinetic‐Pharmacodynamic Framework to Evaluate Bedaquiline Dose Modifications in Adults With Tuberculosis

**DOI:** 10.1002/psp4.70264

**Published:** 2026-05-12

**Authors:** Yu‐Jou Lin, Frances Okibedi, Mats O. Karlsson, Elin M. Svensson

**Affiliations:** ^1^ Department of Pharmacy Uppsala University Uppsala Sweden; ^2^ Department of Clinical Pharmacy & Biochemistry, Institute of Pharmacy Freie Universitaet Berlin Berlin Germany; ^3^ Department of Pharmacy, Pharmacology and Toxicology Radboud University Medical Center Nijmegen the Netherlands

**Keywords:** bedaquiline, dose selection, simulation tool, tuberculosis

## Abstract

Bedaquiline is approved for use in a thrice‐weekly dosing schedule; simpler once‐daily dosing strategies could enable fixed‐dose combinations and improve patient adherence. We developed an interactive R Shiny simulation tool to enable the evaluation of different bedaquiline dosing schedules within an integrated dose‐pharmacokinetic‐efficacy‐safety‐outcome framework. Four models describing bedaquiline and its metabolite M2 concentrations, time‐to‐positivity sputum culture results, QTc intervals, and long‐term outcomes were incorporated in the framework. A virtual population representative of tuberculosis patients was generated for simulation. Two case studies were investigated: (1) alternative bedaquiline dosing regimens and (2) bedaquiline treatment interruption and reinitiation. The ZeNix once‐daily dosing (200 mg once daily for 8 weeks, followed by 100 mg once daily for 16 weeks) yielded 50% lower median bedaquiline and M2 weekly average concentrations compared to the registered (approved) and standard‐loading once‐daily dosing (400 mg once daily for 2 weeks, followed by 100 mg once daily for 22 weeks) at 2 weeks. The exposure difference was predicted to lead to 11% fewer patients with negative cultures at 2 weeks and 5.8% fewer patients having sputum culture conversions at 8 weeks under the ZeNix dosing. The QTcF intervals were predicted from 376 to 442 ms (5th to 95th percentile of the population) for the three evaluated strategies. We also demonstrated that the Shiny application could guide decisions on the timing and doses for reloading bedaquiline after treatment interruption. The developed Shiny platform supports future exploration of bedaquiline treatment under different scenarios, for example, potential drug–drug interactions and clinical trial simulations.

## Study Highlights

- What is the current knowledge on the topic?
  ○ The bedaquiline dose‐exposure‐response relationships for efficacy and safety and how these relate to long‐term outcomes have been established under the registered thrice‐weekly regimen. However, data are limited under simplified once‐daily dosing schedules, which are crucial for improving adherence.
- What question did this study address?
  ○ Whether pharmacokinetic differences due to bedaquiline dose modifications could be clinically relevant regarding efficacy, safety, and long‐term outcomes.
- What does this study add to our knowledge?
  ○ An interactive Shiny application was developed. Our simulation results showed that a standard‐loading once‐daily dosing strategy for bedaquiline (400 mg daily for 2 weeks, then 100 mg daily for 22 weeks) yielded earlier treatment response compared to the ZeNix once‐daily dosing and a similar safety profile compared to the registered dosing. We also demonstrated the use of the app to determine bedaquiline reloading strategies after treatment interruption.
- How might this change clinical pharmacology or translational science?
  ○ The developed simulation tool under an integrated dose‐exposure‐response‐outcome framework allows exploration of bedaquiline treatment under different scenarios. This helps interpret the clinical importance of dose adjustments.

## 1 Introduction

Tuberculosis (TB) remains a serious global health issue. In 2024, approximately 10.8 million people developed TB, and more than 1.25 million people died [1]. Interruption of TB transmission is essential to achieve the targets set in the end TB strategy [1]. Besides the rapid identification of undiagnosed cases and the use of preventive therapy, effective therapy in individuals with active TB disease remains indispensable [2]. Studies have shown that patients receiving prompt treatment rapidly become less infectious, which is important for community‐based infection control [3, 4]. Additionally, intermittent treatment interruption has been found to increase the risk of drug resistance development and poor treatment outcomes [5, 6, 7]. An optimal TB treatment regimen that maximizes patient adherence should be implemented, with clear strategies in place for managing treatment interruptions and re‐initiation [7].

Bedaquiline is a key component in anti‐tuberculosis combination therapy for treating multidrug‐resistant TB (MDR‐TB). Bedaquiline dose‐exposure‐response relationships for efficacy and safety and how these relate to long‐term outcomes have been established under the regulatory‐approved dosing regimen (referred to as registered dosing hereafter) [8, 9, 10, 11]. The registered bedaquiline dosing consists of a 2‐week daily intensive phase and a 22‐week thrice‐weekly continuation phase, which poses challenges for treatment adherence. Simplification to once‐daily dosing is known to improve patient adherence [12, 13]. Moreover, it enables the future development of fixed‐dose combinations, in which bedaquiline can be combined with other daily anti‐TB agents into a single pill to reduce the treatment burden [13, 14]. In 2022, a once‐daily bedaquiline‐containing dosing strategy was recommended by WHO in the approved bedaquiline‐pretomanid‐linezolid with or without moxifloxacin (BPaLM/BPaL) regimen [15, 16]. However, this regimen lacks a higher loading dose in the first 2 weeks, which is important for maximizing early bactericidal activity [17]. An alternative bedaquiline once‐daily dosing strategy, which keeps a 2‐week high loading dose, is currently being tested in the DECISION (NCT05926466) and PARADIGM4TB trial (NCT06114628). Although some pharmacokinetic (PK) simulation studies have compared the PK profiles in different dosing regimens [18, 19], whether the PK differences could be clinically relevant regarding efficacy, safety, and long‐term outcomes remains unclear.

Pharmacometric modeling and simulation techniques serve as powerful tools to quantify dose‐exposure‐response‐outcome relationships [20]. Integrating separate models into a unified framework enables simultaneous evaluation of PK, efficacy, safety, and long‐term treatment outcomes and, therefore, supports dose optimization and decision‐making in protocol development and clinical practice [21, 22]. In addition, web‐based, interactive pharmacometric applications have gained increasing attention recently [23]. The Shiny package for R allows end‐users to interactively explore complex results without requiring extensive knowledge of programming or modeling. This enhances the visualization of outputs from the models and facilitates communication among researchers, clinicians, policy makers, and patient representatives, thereby promoting wider accessibility and engagement with pharmacometrics for a broad audience [24, 25]. In turn, it enables evaluation of trade‐offs and exploration of scenarios relevant for both decision‐makers and patients. In this work, we aimed to demonstrate the use of an interactive R Shiny simulation tool to evaluate bedaquiline dosing modifications within an integrated dose‐PK‐efficacy‐safety‐outcome modeling framework, exemplified by two case studies: (1) comparison of the registered thrice‐weekly dosing and alternative once‐daily dosing regimens, and (2) evaluation of strategies for bedaquiline dose interruptions and reinitiation. While the framework provides insights into these specific exploratory scenarios, this tool is intended for exploring a broad range of dosing strategies under the models' underlying assumptions, provided that results are interpreted with caution when applied to entirely novel regimens or populations.

## 2 Methods

### 2.1 Integrated Modeling Framework

**FIGURE 1:**
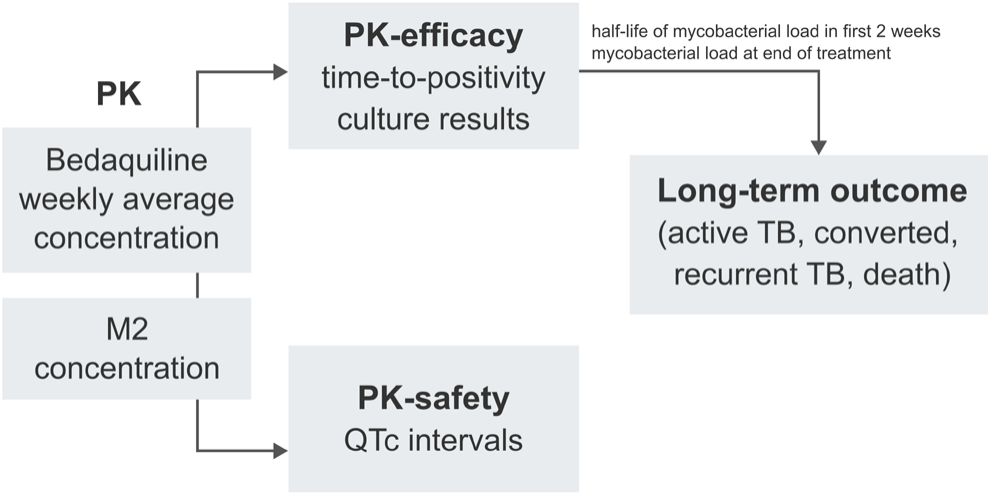
Integrated bedaquiline dose‐PK‐efficacy‐safety‐outcome modeling framework for adult patients with tuberculosis. The time‐to‐positivity results from MGIT liquid cultures were predicted by weekly average concentration of bedaquiline, and the QTc interval was predicted by M2 concentration (bedaquiline's main metabolite). Two on‐treatment biomarkers derived from the PK‐efficacy model (half‐life of mycobacterial load in the first 2 weeks and mycobacterial load at the end of treatment) were used to predict long‐term treatment outcomes (active TB, converted, recurrent TB, and death). MGIT, mycobacteria growth indicator tube; PK, pharmacokinetics; QTc, corrected QT; TB, tuberculosis.

Four developed models were incorporated in the framework (Figure 1): a population PK model characterizing concentrations of bedaquiline and its main metabolite M2 [8], a PK‐efficacy model [9], a PK‐safety model [10], and a long‐term TB treatment outcome model [11]. Within this framework, the PK‐efficacy model described how the weekly average concentration of bedaquiline drove changes in mycobacterial load in patients and, therefore, the time‐to‐positivity (TTP) values and probability of having a positive sputum culture result in the mycobacteria growth indicator tube (MGIT) liquid culture system. The PK‐safety model characterized the relationship between M2 concentrations and corrected‐QT (QTc) prolongation, the primary safety concern in bedaquiline treatment. In the multistate outcome model, two covariates derived from the PK‐efficacy model, namely half‐life of mycobacterial load at the first 2 weeks and mycobacterial load at the end of treatment, were identified as on‐treatment predictors of conversion and recurrence, respectively. Other covariates in the framework included age, biological sex (male/female), race (black/non‐black), baseline albumin‐corrected calcium and potassium measurements, co‐administration with clofazimine or moxifloxacin (Yes/No), baseline TTP in MGIT cultures, and time‐varying weight and albumin concentration. The drug–drug interaction effect when co‐administered with other anti‐TB agents (rifampicin, rifapentine) and antiretroviral medications (efavirenz, lopinavir/ritonavir, and nevirapine) was incorporated in the bedaquiline PK model [26, 27, 28]. The models implemented in the framework were verified by comparing the key figures from publications and the outputs from the recoded models.

Given the changes in the definition of drug‐resistant TB and the evolution of background regimens over the past two decades [29], mycobacterial killing is thought to be more rapid than it was during the clinical trials on which the models were based. To align with the current TB treatment setting, the half‐life of mycobacterial load decline in the PK‐efficacy model is allowed to be adjusted according to the expected conversion rate under the given TB combination therapy. Together with the variability of half‐life described in the PK‐efficacy model, this is deemed to capture the variations in response across different resistance levels and background regimens, allowing for a more consistent evaluation of different bedaquiline regimens. The expected conversion rate at 2 months and 6 months over a range of half‐life values was derived from the simulated individual TTP data using the PK‐efficacy model (Figure S1). The reference half‐life of mycobacterial load in 0.54 weeks represents the value reported in the developed model using patient data under 24‐week registered doses of bedaquiline on top of a five‐drug background regimen including ethionamide, pyrazinamide, ofloxacin, kanamycin, and cycloserine from 2007 to 2008.

### 2.2 Virtual Population

A large virtual population of 8340 individuals (15‐fold of the pooled original data) with baseline covariate distributions representative of TB patients was generated using conditional distribution modeling [30], based on data from 556 patients pooled from three TB clinical trials (two phase IIb trials [NCT01498419, NCT02193776] and one phase III trial [NCT02333799]) available on the TB‐PACTS platform [31]. Each covariate value was simulated conditionally based on the observed or imputed values from other covariates using multivariate imputation by chained equations (MICE). This process was repeated iteratively until convergence, meaning that the imputed covariate values stabilized across iterations. Time‐varying weight and albumin concentration were simulated from the population PK model of bedaquiline and M2 [8], given the imputed baseline values from conditional distribution modeling.

### 2.3 R Shiny Application and Relevant Packages

All work was performed in R (version 4.4.1) with relevant packages for data processing, simulation and visualization. The virtual TB population was generated using the *mice* package (version 3.16.0). All simulations were performed using the *mrgsolve* package (version 1.5.1). Specifically, PK and PK‐safety simulations and results were run and visualized in the R Shiny platform (version 1.9.1). PK‐efficacy and outcome simulation results were processed locally in R, as the developed Shiny app only supports a single simulation at a time but cannot summarize over a number of simulations, which were needed for reducing random noise in probabilistic models. The developed Shiny application is available at: https://bedaquiline‐dose‐pk‐pd‐outcome.serve.scilifelab.se. The source code, data and required packages to reproduce the results can be accessed on GitHub: https://github.com/slove1200/BDQ‐simulation‐framework‐shiny.

### 2.4 Simulation Settings for Case Study 1—Bedaquiline Dose Evaluation

Three bedaquiline dosing regimens were evaluated: the registered dosing (400 mg once daily for 2 weeks, then 200 mg thrice weekly for 22 weeks), the ZeNix once‐daily dosing (200 mg once daily for 8 weeks, then 100 mg once daily for 16 weeks), the alternative standard‐loading once‐daily dosing (400 mg once daily for 2 weeks, then 100 mg once daily for 22 weeks). Simulations were performed for 500 patients sampled from the virtual population for each regimen. Individuals were sampled with a proportion of 50%/50% male and female, and 40% were assumed to be of black race. The same 500 sampled individuals were used across the three regimens to enable the direct comparison of all aspects in the framework. The default value of half‐life of mycobacterial load decline was set to be 0.33 weeks to match the observed 80% and 95% conversion rate at 2 months and 6 months in the recent Nix‐TB trial [32].

Simulations from the PK‐efficacy and long‐term outcome models were performed 100 times to diminish the stochastic variability and ensure that the difference in results observed between regimens is attributed to dosing schedules rather than random noise. The results simulated from the PK‐efficacy and outcome models were summarized into 5th, median, and 95th percentiles in the 100 times of simulations per regimen. The simulated PK concentration and QTc level over time were summarized into 5th, median, and 95th percentiles across the population per regimen. The simulation period was 24 weeks for PK, efficacy, and safety, and 120 weeks for long‐term outcomes.

### 2.5 Simulation Settings for Case Study 2—Bedaquiline Treatment Interruption and Reinitiation

In this case example, we set up a total of 9 scenarios representing all combinations of three interruption conditions (no interruption, 8 weeks of interruption without or with reloading bedaquiline at 12 weeks of treatment) and three regimens (registered, ZeNix, and standard‐loading once‐daily dosing). The reloading dosing strategies tested in the case example were based on a previous simulation study on optimizing the reloading bedaquiline strategy [33]: 400 mg once daily for 7 days for the registered and standard‐loading once‐daily regimens, and 200 mg once daily for 21 days for the ZeNix regimen. The total treatment duration was 24 weeks in all scenarios. Since the purpose of the case study is to showcase one example of utilizing the Shiny app, a typical individual (32‐year‐old non‐black male weighing 56.6 kg, with baseline albumin 3.65 g/dL, albumin‐corrected calcium 2.44 mmol/L, potassium 4.2 mmol/L) instead of a population was chosen for simplicity. The efficacy and treatment outcomes were not assessed in this case, as the models were developed using clinical trial data in which the bedaquiline treatment was not interrupted for long intervals and were not fully mechanistic; therefore, several factors, such as the development of resistance and shifts in mycobacterial subpopulations, cannot be captured by the models. The simulation period for PK and safety was set to be 32 weeks, that is, a maximum treatment plus interruption period in the investigated scenarios.

## 3 Results

### 3.1 R Shiny Application Workflow

**FIGURE 2:**
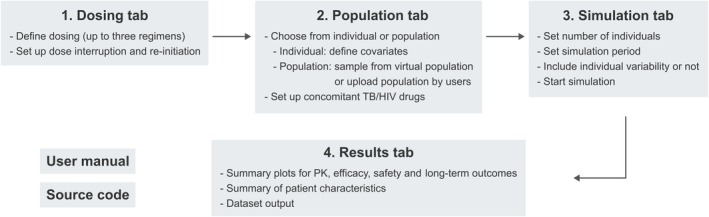
Workflow of the Shiny app for evaluating bedaquiline dose modifications. In the dosing tab (Step 1), users define up to three dosing regimens with or without interruption scenarios. In the population tab (Step 2), users choose to simulate at an individual or population level and specify covariates. If the population level is selected, users can choose to use the virtual population or upload custom population data. The simulation tab (Step 3) allows users to specify the number of individuals and duration for simulation, and whether to include interindividual variability before initiating simulations. Outputs are displayed in the results tab (Step 4), including PK profiles, efficacy, safety, long‐term outcomes, and summary statistics for patient characteristics. The app also provides the user manual and source code for step‐by‐step guidance and reproducibility. The Shiny app is available at https://bedaquiline‐dose‐pk‐pd‐outcome.serve.scilifelab.se/, and the source code can be accessed on GitHub (https://github.com/slove1200/BDQ‐simulation‐framework‐shiny). HIV, human immunodeficiency virus; PK, pharmacokinetics; TB, tuberculosis.

Figure 2 illustrates the R Shiny app workflow. In the “1. Dosing” tab, users define bedaquiline dosing schedules by specifying loading and maintenance doses, including the optional treatment interruptions. Up to three regimens can be added and compared. In the “2. Population” tab, users can select to simulate either an individual or a virtual population, and values for range and proportion of covariates can be specified. This section also includes options for setting the half‐life of mycobacterial load decline and adding concomitant medications that influence PK or QT levels. The “3. Simulation” tab allows the configuration of key simulation parameters, such as the number of individuals, simulation duration, and whether to include interindividual variability. Once all the settings are completed, users can click “start simulation”. All the simulation results are generated and displayed in the “4. Results” tab, where users can explore simulated PK profiles, efficacy (MGIT sputum culture results), safety (QTc intervals using the Fridericia formula, that is, QTcF), and long‐term treatment outcomes. All the data outputs can be exported as CSV files with accompanying data specifications, while the generated graphs can be visualized and saved directly from the app via right‐click. The introduction and step‐by‐step instructions of the app can be found in the “About–User Manual” tab. The source code is provided along with installation instructions in the “About–Source Code” tab for users to run the app locally, which is needed for simulations with a large number of individuals. The app also offers an additional module for simulating individual TTP data, accessible via the “Extra: TTP Simulation” tab (detailed in the Supporting Information).

### 3.2 Case Study 1: Evaluation of Bedaquiline Dosing Strategies

**FIGURE 3:**
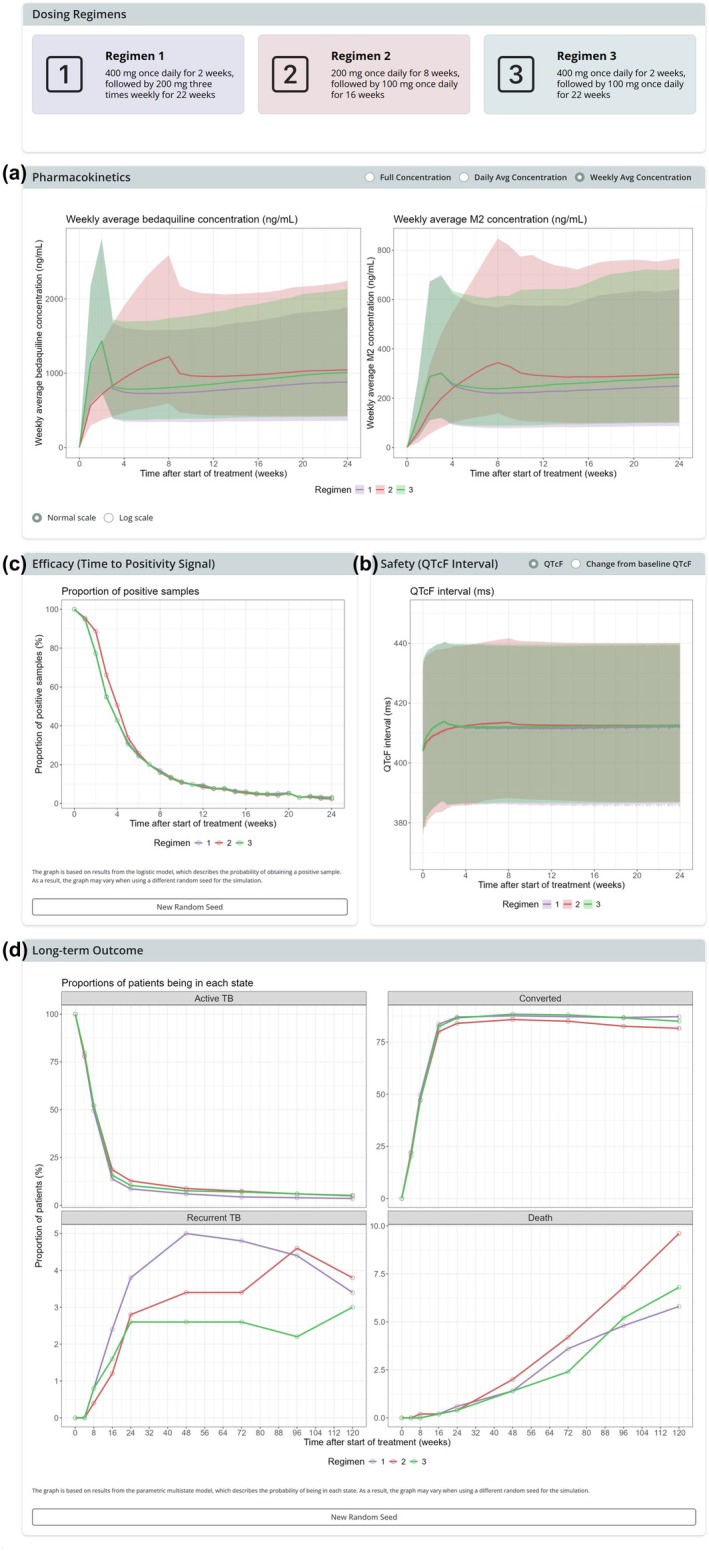
Simulation results displayed in the Shiny app interface under the registered dosing (Regimen 1, purple), the ZeNix once‐daily dosing (Regimen 2, red), and the standard‐loading once‐daily dosing (Regimen 3, green) for bedaquiline. Simulations were performed for 500 individuals sampled from a representative TB virtual population with a proportion of 50%/50% male and female and assumed to be of 40% black race. Predicted (a) weekly average concentration of bedaquiline and bedaquiline's main metabolite M2, (b) QTcF intervals, and (c) proportion of positive MGIT cultures during the 24‐week treatment period. (d) Predicted proportion of patients being in active TB, converted, recurrent TB and death states over 120 weeks. For plots of (a) concentrations and (b) QTcF intervals, solid lines represent the medians, and the shaded areas represent the 5th to 95th percentiles of the population. Noted that (c) efficacy and (d) outcome results were generated with a specific random seed; results may differ if a new random seed is selected. QTcF, Fridericia‐corrected QT intervals; TB, tuberculosis.

**FIGURE 4:**
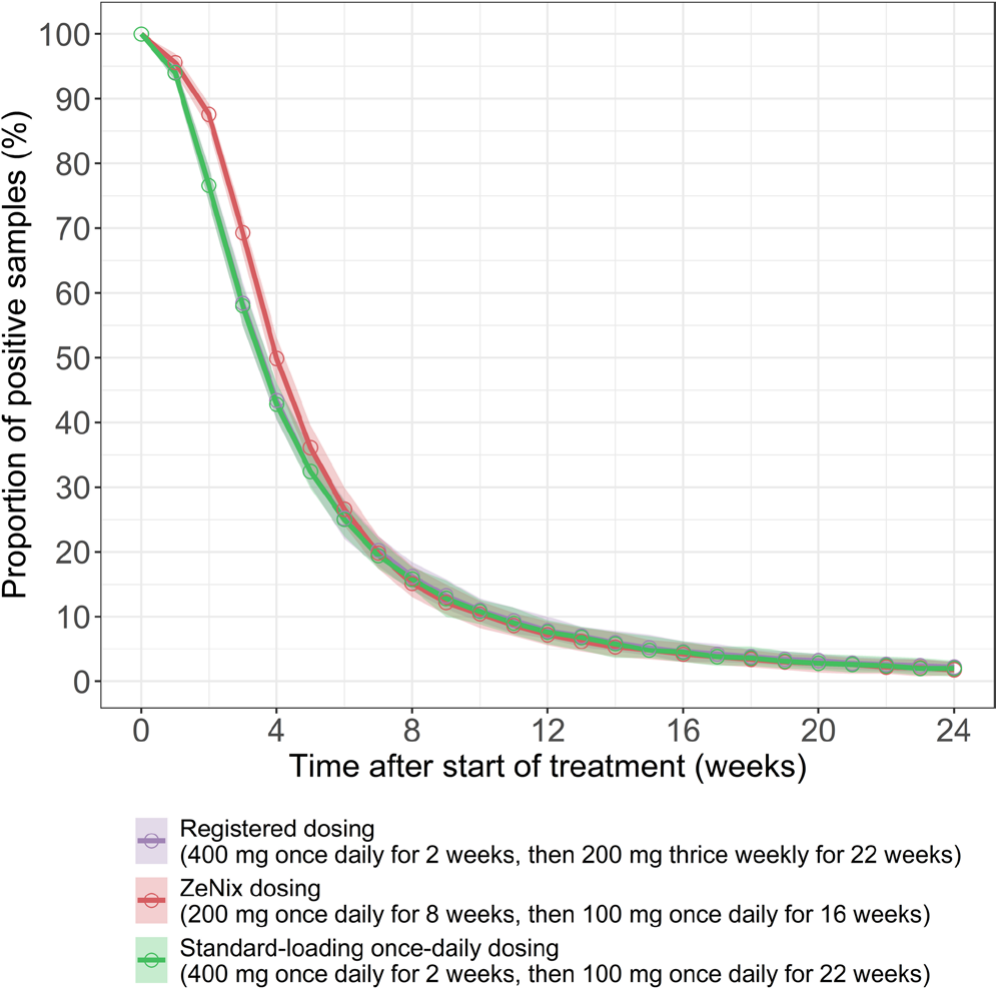
The predicted proportion of positive MGIT liquid cultures during the 24‐week treatment period over 100 times of simulation under the registered dosing (purple), the ZeNix once‐daily dosing (red), and the standard‐loading once‐daily dosing (green) for bedaquiline. Solid lines represent the medians, and the shaded areas indicate the 5th to 95th percentiles from 100 simulations. Noted that the purple line and its shaded area (registered dosing) are behind the green (standard‐loading once‐daily dosing). MGIT, mycobacteria growth indicator tube.

**FIGURE 5:**
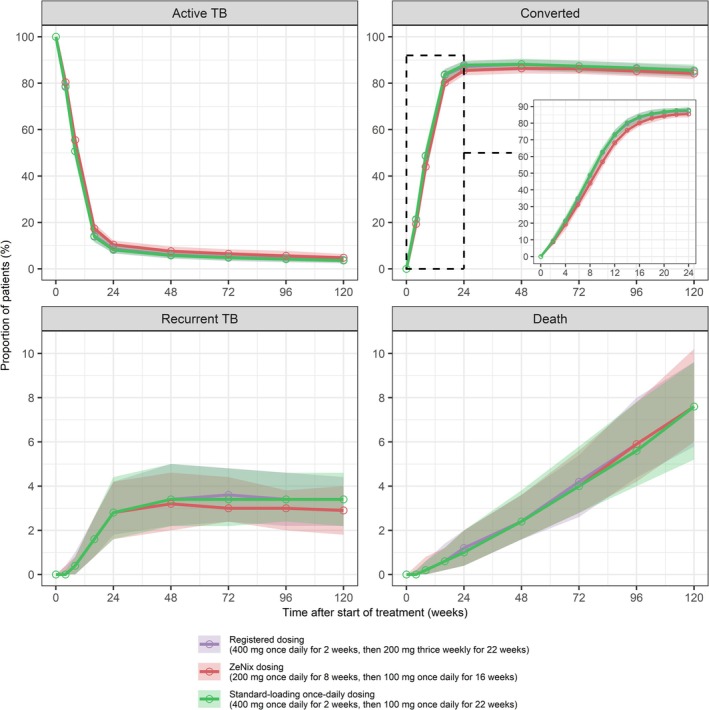
The predicted proportion of patients being in active TB, converted, recurrent TB and death states in 120 weeks over 100 times of simulation under the registered dosing (purple), the ZeNix once‐daily dosing (red), and the standard‐loading once‐daily dosing (green) for bedaquiline. The inset in the converted panel displays the early treatment phase (0–24 weeks) to highlight differences in the converted state across regimens. Solid lines represent the medians, and the shaded areas indicate the 5th–95th percentiles from 100 simulations. Noted that the purple line and its shaded area (registered dosing) are behind the green (standard‐loading once‐daily dosing). TB, tuberculosis.

The characteristics of the 500 patients sampled from the virtual population are summarized in Figures S2 and S3. Figure 3a displays the weekly average concentration of bedaquiline and M2 over the 24‐week treatment period for each evaluated regimen. Compared to the registered dosing and standard‐loading once‐daily dosing, the median bedaquiline cumulative AUCs were predicted to be 50% and 26% lower under ZeNix dosing at 2 and 4 weeks, respectively. By 8 weeks, the median cumulative AUCs of bedaquiline were comparable across three regimens. Predicted QTcF intervals ranged from 376 to 442 ms (5th–95th percentile of the population), which were under the safety threshold of 500 ms for all three evaluated dosing strategies (Figure 3b). A single simulation example of efficacy and outcome results generated in the Shiny app is illustrated in Figure 3c,d. Due to the stochastic nature of the efficacy and outcome models, the output of a single simulation is dependent on the specific random seed and each execution is expected to generate slightly different results. To reduce the stochastic randomness in the simulation and evaluate the regimen differences robustly, 100 simulations were performed. The predicted proportion of patients having negative sputum cultures was comparable throughout the treatment period for the registered and standard‐loading once‐daily regimens, whereas the ZeNix dosing yielded 11% fewer patients with negative sputum culture at 2 weeks (Figure 4). The difference in the proportion of culture results between ZeNix and the other two dosing strategies diminished over time after the first month. A similar trend was observed in the predicted treatment outcomes (Figure 5), with the proportion of patients in the converted state reflecting the differences in negative sputum culture samples. At 8 weeks, the ZeNix dosing resulted in 5.8% fewer patients having sputum culture conversions compared to the registered and standard‐loading once‐daily dosing schedules. However, the proportion of patients with recurrence or death remained similar across all regimens over 120 weeks.

### 3.3 Case Study 2: Bedaquiline Reinitiation After Interruption

**FIGURE 6:**
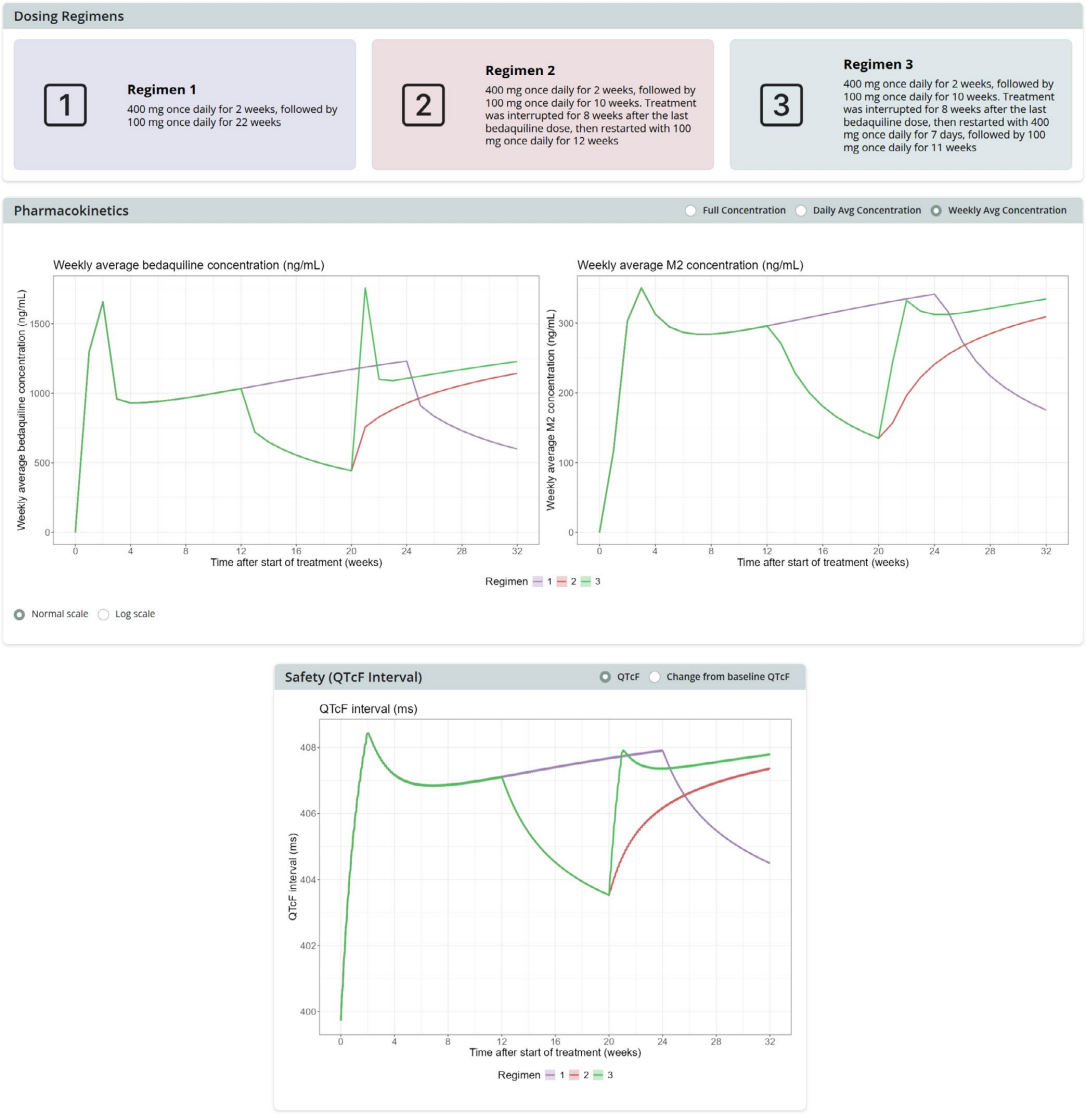
Evaluation of reloading strategies with interruption under the standard‐loading once‐daily dosing regimen for bedaquiline in the Shiny interface app. Regimen 1 (purple): Bedaquiline treatment for 24 weeks without interruption. Regimen 2 (red): 8 weeks of interruption after 12 weeks of bedaquiline therapy, and then reinitiated bedaquiline treatment without reloading doses. Regimen 3 (green): 8 weeks of interruption after 12 weeks of bedaquiline therapy, and then reinitiated bedaquiline therapy with a 400 mg daily dose for 7 days. Simulations were performed for a typical individual (32‐year‐old non‐black male weighing 56.6 kg, with baseline albumin 3.65 g/dL, albumin‐corrected calcium 2.44 mmol/L, potassium 4.2 mmol/L).

The second case was to demonstrate how to use the Shiny app to simulate specific interruption scenarios and evaluate optimal restarting doses for bedaquiline. Figure 6 illustrates the three scenarios of bedaquiline reinitiation strategies after treatment interruption under the standard‐loading once‐daily dosing regimen. To quickly reach comparable exposures under the interruption of 8 weeks after 12 weeks of bedaquiline treatment, a 400‐mg loading dose for 7 days was needed to restore adequate bedaquiline exposure without the concern of QTc prolongation. Other scenarios with the registered dosing and ZeNix dosing strategies are shown in Figures S4 and S5.

## 4 Discussion

We developed a modeling framework to simultaneously describe the dose‐exposure‐response‐outcome relationship of bedaquiline treatment, which is implemented in the web‐based R Shiny application. In the first case study, three dosing strategies (the registered thrice‐weekly dosing, the once‐daily ZeNix dosing, and the standard‐loading once‐daily dosing) were compared. The simulated median weekly average concentration and cumulative AUC of bedaquiline and M2 aligned with the previously published simulation studies [18, 19]. Our simulations demonstrated that the low early exposure of bedaquiline under the ZeNix dosing is expected to lead to a reduced proportion of patients achieving negative sputum cultures compared to registered and standard‐loading once‐daily dosing, particularly in the first 2 months. The predicted percentage of patients having sputum culture conversion reflected the difference in early treatment response across three regimens. However, our simulation results showed no substantial differences in recurrence or mortality outcomes across the evaluated dosing strategies, despite end‐of‐treatment mycobacterial load being a significant predictor of recurrence in the model [11]. Compared to the approved dosing, median M2 concentrations were 56% and 19% higher under the ZeNix dosing (8.4% and 14% higher under standard‐loading once‐daily dosing) at 8 and 24 weeks, respectively. Nevertheless, the predicted 95th percentile of QTcF intervals was below the 500 ms threshold with no substantial difference for all three evaluated dosing strategies, suggesting minimal safety concern under both once‐daily regimens.

Early halt of TB transmission is crucial to reduce TB incidence [3, 34]. Several studies have highlighted the importance of effective treatment in stopping TB transmission: experiments on human‐to‐guinea pig transmission showed that adequate treatment stops transmission almost immediately [35, 36], while a cough aerosol study found that effective treatment led to a rapid decline in mycobacterial counts in the first 3 weeks, suggesting that patients become less likely to transmit TB during the early course of therapy [37]. In addition, successful treatment greatly relieved the economic burden for TB patients and their families [38]. One important attribute of treatment success is the improvement of patient adherence [39, 40]. A cost‐effectiveness study on novel tuberculosis treatment regimens showed that, in addition to shortening treatment duration, patient‐friendly regimens could substantially reduce the economic burden on patients and their families [39]. Our simulation findings indicated that the standard‐loading once‐daily bedaquiline regimen may outperform the ZeNix dosing with respect to early transmission interruption while offering better adherence than the currently approved thrice‐weekly dosing.

Optimization of restarting doses for bedaquiline is one of the interests in the current TB treatment. Given the extremely long terminal half‐lives of bedaquiline and M2, patients can have remaining bedaquiline and M2 concentrations in the body years after treatment interruptions. Selecting an appropriate strategy for reloading bedaquiline doses can help restore adequate exposures as quickly as possible while minimizing the risk of QTc prolongation. Our developed Shiny app serves as a valuable tool for evaluating reinitiation strategies in a flexible and easy‐to‐use way. Simulated drug concentration and QTc profiles for individuals with specific characteristics under different dosing schedules and interrupted intervals can be visualized to further support evidence‐based decisions.

In addition to the simulation studies evaluating bedaquiline dose modifications in this work, the developed Shiny app can be utilized for other purposes. For example, the impact of drug–drug interactions in the aspects of PK profiles and QTc prolongation can be explored and visualized with just a few clicks. The predicted curves can be easily visualized under specified dosing schedules and facilitate clinical decision‐making. Additionally, the individual TTP data can be simulated with information on user‐defined culture sampling timepoints, distribution of baseline TTP for individuals, and half‐life of mycobacterial load decline. The output can be used for further analyses of the TTP data, for example, power calculations (see details in the Supporting Information). The Shiny app provides an easy‐to‐use platform for experts from various fields, including not only clinicians and policy‐makers but also other stakeholders. Given the open‐source property of the Shiny app, users can adjust and customize the source codes by themselves and run the program locally, allowing for a flexible simulation environment. Future perspective for the Shiny simulation tool includes extension from adults to pediatric population to allow a broader application in evaluating bedaquiline dosing strategies across weight and age groups.

Several assumptions and limitations were present in this study. The half‐life of mycobacterial load decline is a latent variable derived from the PK‐efficacy model used in the framework. The half‐life value cannot be directly translated into other bacterial killing parameters [41, 42, 43], for example, rate of change in TTP, as they may not reflect the same extent of bacterial killing rate. Due to the data source used in the PK‐efficacy model development, the exposure‐response relationship cannot be extrapolated beyond the treatment period or in the interruption and reinitiation cases. Similarly, the developed outcome model does not support the assessment of different ranges of bedaquiline treatment duration other than the standard 24 weeks. Lastly, in the simulation framework, all patients were assumed to have similar adherence to the medications observed in the clinical trials, which may not be valid in real‐world settings. Such adherence patterns can be modeled as a covariate effect on exposures or through a Markov chain with probabilities of missing a dose [44, 45], providing a more realistic representation of patient behaviors in clinical trial design. Although adherence was not explicitly modeled, the association between simplified regimens and higher adherence [12, 13] suggested no worse adherence with once‐daily dosing than with standard thrice‐weekly dosing, which likely led to marginally higher exposures of bedaquiline and M2.

To conclude, we developed a simulation framework incorporating a PK model, two exposure‐response models for sputum culture results as well as QTc intervals, and a long‐term outcome model for patients under bedaquiline treatment. Our case study showed that the early treatment response under the ZeNix dosing differed from the registered and standard‐loading once‐daily dosing strategies. The standard‐loading once‐daily dosing showed the advantages of earlier sputum culture conversion. Additionally, QTc intervals were within safe limits across all regimens. This suggests that the standard‐loading once‐daily dosing strategy is both efficacious and safe. The developed Shiny platform can be used for future exploration of bedaquiline treatment under different scenarios, for example, potential drug–drug interactions and clinical trial simulations. The source code and simulation outputs are available for download, allowing users to perform computationally intensive simulations locally and conduct any statistical tests of interest on the results. This enables easy and quick exploration of bedaquiline dosing and helps interpret the clinical importance of dose adjustments.

## Author Contributions

Yu‐Jou Lin, Frances Okibedi, Mats O. Karlsson, and Elin M. Svensson wrote the manuscript. Yu‐Jou Lin, Frances Okibedi, Mats O. Karlsson, and Elin M. Svensson designed the research. Yu‐Jou Lin and Frances Okibedi performed the research. Yu‐Jou Lin, Mats O. Karlsson, and Elin M. Svensson analyzed the data.

## Funding

This project was funded from the Innovative Medicines Initiative 2 Joint Undertaking (JU) under grant agreement No 101007873. The JU receives support from the European Union's Horizon 2020 research and innovation programme and EFPIA, Deutsches Zentrum für Infektionsforschung e. V. (DZIF), and Ludwig‐Maximilians‐Universität München (LMU). EFPIA/AP contribute to 50% of funding, whereas the contribution of DZIF and the LMU University Hospital Munich has been granted by the German Federal Ministry of Education and Research.

## Conflicts of Interest

Elin M. Svensson and Mats O. Karlsson have previously received research grants from Janssen Pharmaceuticals, the developer of bedaquiline. Yu‐Jou Lin and Frances Okibedi declare no conflicts of interest.

## Supporting information


**Figure S1:** Predicted conversion rate over half‐life of mycobacterial load decline in patients with multidrug‐resistant tuberculosis. The solid lines represent the conversion rate at Month 2 (red) and Month 6 (blue) over various half‐life of mycobacterial load decline. The dashed vertical line represents the half‐life reported in the developed model with 24‐week registered bedaquiline treatment on top of a five‐drug background regimen including ethionamide, pyrazinamide, ofloxacin, kanamycin, and cycloserine. Given the reported half‐life (0.54 weeks), the predicted Month 2 and 6 conversion rates were 56.1% and 89.1%, respectively. This could help users select the most suitable value of half‐life in the current tuberculosis treatment settings.
**Figure S2:** Screenshot of the summary table of baseline characteristics for the sampled 500 patients in case study 1 on bedaquiline dose evaluation. Corrected calcium level is calculated using the following equation: corrected calcium level (mmol/L) = measured calcium level (mmol/L) + 0.8 × (4 − albumin level (g/dL)).
**Figure S3:** Screenshot of the summary plots of baseline continuous covariates for the sampled 500 patients compared to the large virtual population. The boxplots represent the covariate distribution in the large virtual population using conditional distribution modeling. The dots represent the sampled 500 patients in case study 1 on bedaquiline dose evaluation. Corrected calcium level is calculated using the following equation: corrected calcium level (mmol/L) = measured calcium level (mmol/L) + 0.8 × (4 − albumin level (g/dL)). Ca, calcium; TTP, timeto‐positivity.
**Figure S4:** Evaluation of reloading strategies with interruption under the regulatory‐approved thrice‐weekly dosing regimen for bedaquiline in the Shiny interface app. Regimen 1 (purple): bedaquiline treatment for 24 weeks without interruption. Regimen 2 (red): 8 weeks of interruption after 12 weeks of bedaquiline therapy, and then reinitiated bedaquiline treatment without reloading doses. Regimen 3 (green): 8 weeks of interruption after 12 weeks of bedaquiline therapy, and then reinitiated bedaquiline therapy with a 400 mg daily dose for 7 days. Simulations were performed for a typical individual (32‐year‐old non‐black male weighing 56.6 kg, with baseline albumin 3.65 g/dL, albumin‐corrected calcium 2.44 mmol/L, potassium 4.2 mmol/L).
**Figure S5:** Evaluation of reloading strategies with interruption under the ZeNix once‐daily dosing regimen for bedaquiline in the Shiny interface app. Regimen 1 (purple): bedaquiline treatment for 24 weeks without interruption. Regimen 2 (red): 8 weeks of interruption after 12 weeks of bedaquiline therapy, and then reinitiated bedaquiline treatment without reloading doses. Regimen 3 (green): 8 weeks of interruption after 12 weeks of bedaquiline therapy, and then reinitiated bedaquiline therapy with a 200 mg daily dose for 21 days. Simulations were performed for a typical individual (32‐year‐old non‐black male weighing 56.6 kg, with baseline albumin 3.65 g/dL, albumin‐corrected calcium 2.44 mmol/L, potassium 4.2 mmol/L).
**Figure S6:** Screenshot of the Shiny interface to simulate individual time‐to‐positivity (TTP) data. The simulation tool allows users to define simulation settings, including baseline TTP distribution, half‐life of mycobacterial load decline, culture sampling schedule, number of culture replicates, and definition of sputum culture conversion (left panel). The simulation outputs include individual‐level TTP data (top right) and a time to sputum culture conversion plot (bottom right, only applicable when culture sampling time unit is in weeks). A CSV file of the simulation dataset together with a data specification text file can be downloaded by clicking “Download TTP data”.
