# Supplementary material for: An Interactive Pharmacokinetic‐Pharmacodynamic Framework to Evaluate Bedaquiline Dose Modifications in Adults With Tuberculosis
Source: CPT Pharmacometrics Syst Pharmacol. 2026 May 12;15(5):e70264. doi: 10.1002/psp4.70264 (PMC13274661; doi:10.1002/psp4.70264)
Supplement: Supplementary file 1 — Figure S1: Predicted conversion rate over half‐life of mycobacterial load decline in patients with multidrug‐resistant tuberculosis. The solid lines represent the conversion rate at Month 2 (red) and Month 6 (blue) over various half‐life of mycobacterial load decline. The dashed vertical line represents the half‐life reported in the developed model with 24‐week registered bedaquiline treatment on top of a five‐drug background regimen including ethionamide, pyrazinamide, ofloxacin, kanamycin, and cycloserine. Given the reported half‐life (0.54 weeks), the predicted Month 2 and 6 conversion rates were 56.1% and 89.1%, respectively. This could help users select the most suitable value of half‐life in the current tuberculosis treatment settings. Figure S2: Screenshot of the summary table of baseline characteristics for the sampled 500 patients in case study 1 on bedaquiline dose evaluation. Corrected calcium level is calculated using the following equation: corrected calcium level (mmol/L) = measured calcium level (mmol/L) + 0.8 × (4 − albumin level (g/dL)). Figure S3: Screenshot of the summary plots of baseline continuous covariates for the sampled 500 patients compared to the large virtual population. The boxplots represent the covariate distribution in the large virtual population using conditional distribution modeling. The dots represent the sampled 500 patients in case study 1 on bedaquiline dose evaluation. Corrected calcium level is calculated using the following equation: corrected calcium level (mmol/L) = measured calcium level (mmol/L) + 0.8 × (4 − albumin level (g/dL)). Ca, calcium; TTP, timeto‐positivity. Figure S4: Evaluation of reloading strategies with interruption under the regulatory‐approved thrice‐weekly dosing regimen for bedaquiline in the Shiny interface app. Regimen 1 (purple): bedaquiline treatment for 24 weeks without interruption. Regimen 2 (red): 8 weeks of interruption after 12 weeks of bedaquiline therapy, and then reinitiated bedaquiline t [file PSP4-15-e70264-s001.pdf]

Supplementary Material

An interactive pharmacokinetic-pharmacodynamic framework to evaluate bedaquiline dose modifications in adults with tuberculosis

Yu-Jou Lin[1], Frances Okibedi[1,2], Mats O. Karlsson[1], Elin M. Svensson[1,3]

[1]Department of Pharmacy, Uppsala University, Uppsala, Sweden

[2]Department of Clinical Pharmacy & Biochemistry, Institute of Pharmacy, Freie Universitaet Berlin, Berlin, Germany

[3]Department of Pharmacy, Pharmacology and Toxicology, Radboud University Medical Center, Nijmegen, The Netherlands

Correspondence:

Elin M. Svensson

Address: Department of Pharmacy, Uppsala University, P.O. Box 580, 751 23 Uppsala, Sweden

Email: elin.svensson@uu.se

Table of Contents

Supplementary Figures 3

Additional feature in the Shiny application: Simulation of time to positivity (TTP) data 8

Supplementary Figures

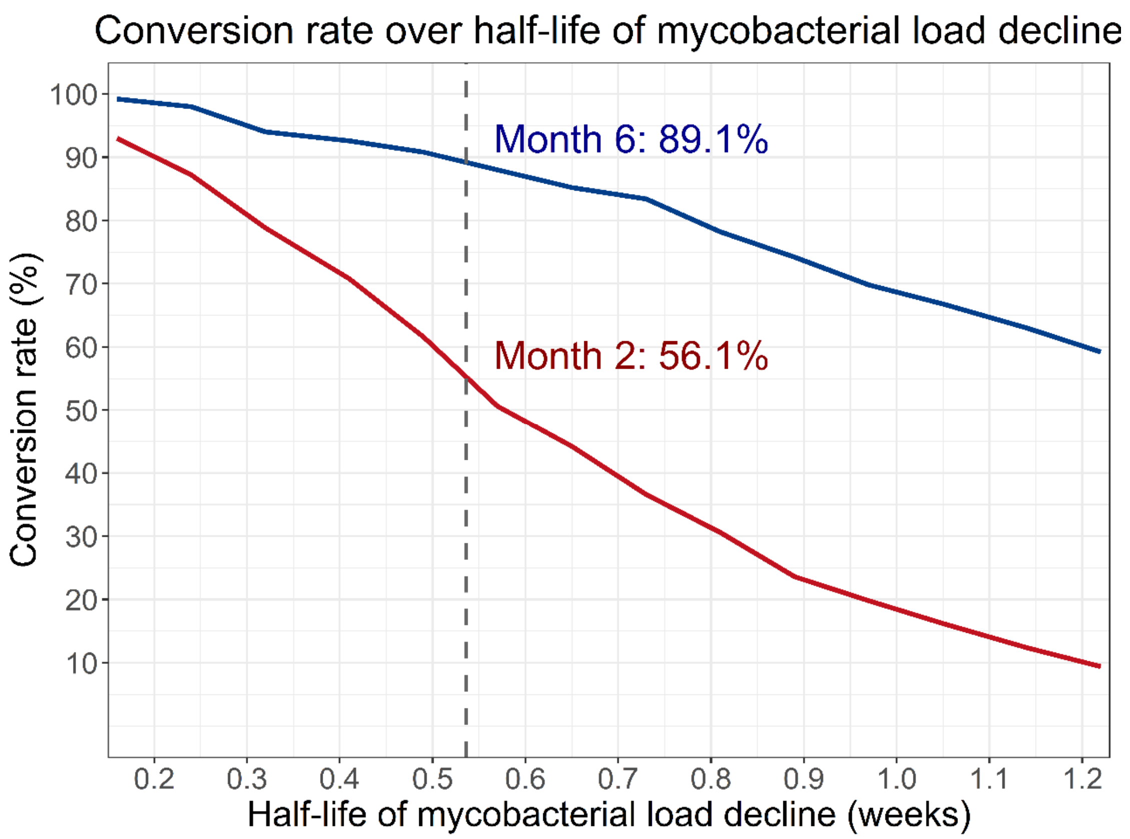

Figure S1. Predicted conversion rate over half-life of mycobacterial load decline in patients with multidrug-resistant tuberculosis. The solid lines represent the conversion rate at month 2 (red) and month 6 (blue) over various half-life of mycobacterial load decline. The dashed vertical line represents the half-life reported in the developed model with 24-week registered bedaquiline treatment on top of a five-drug background regimen including ethionamide, pyrazinamide, ofloxacin, kanamycin, and cycloserine. Given the reported half-life (0.54 weeks), the predicted month 2 and month 6 conversion rates were 56.1% and 89.1%, respectively. This could help users select the most suitable value of half-life in the current tuberculosis treatment settings.

Table

Variable	Median (Range) or N (%)
**Patient Characteristics**	
Age (years)	30 (17-69)
Weight (kg)	53.1 (33-90)
Sex (female)	250 (50%)
Race (black)	200 (40%)
**Laboratory Values**	
Albumin (g/dL)	3.5 (2-5)
Corrected calcium level (mmol/L)	2.38 (2-3.08)
Potassium level (mmol/L)	4.5 (3.4-6.26)
Baseline time-to-positivity (days)	5.08 (2.25-42)

Figure S2. Screenshot of the summary table of baseline characteristics for the sampled 500 patients in case study 1 on bedaquiline dose evaluation. Corrected calcium level is calculated using the following equation: corrected calcium level (mmol/L) = measured calcium level (mmol/L) + 0.8 × (4 − albumin level (g/dL)).

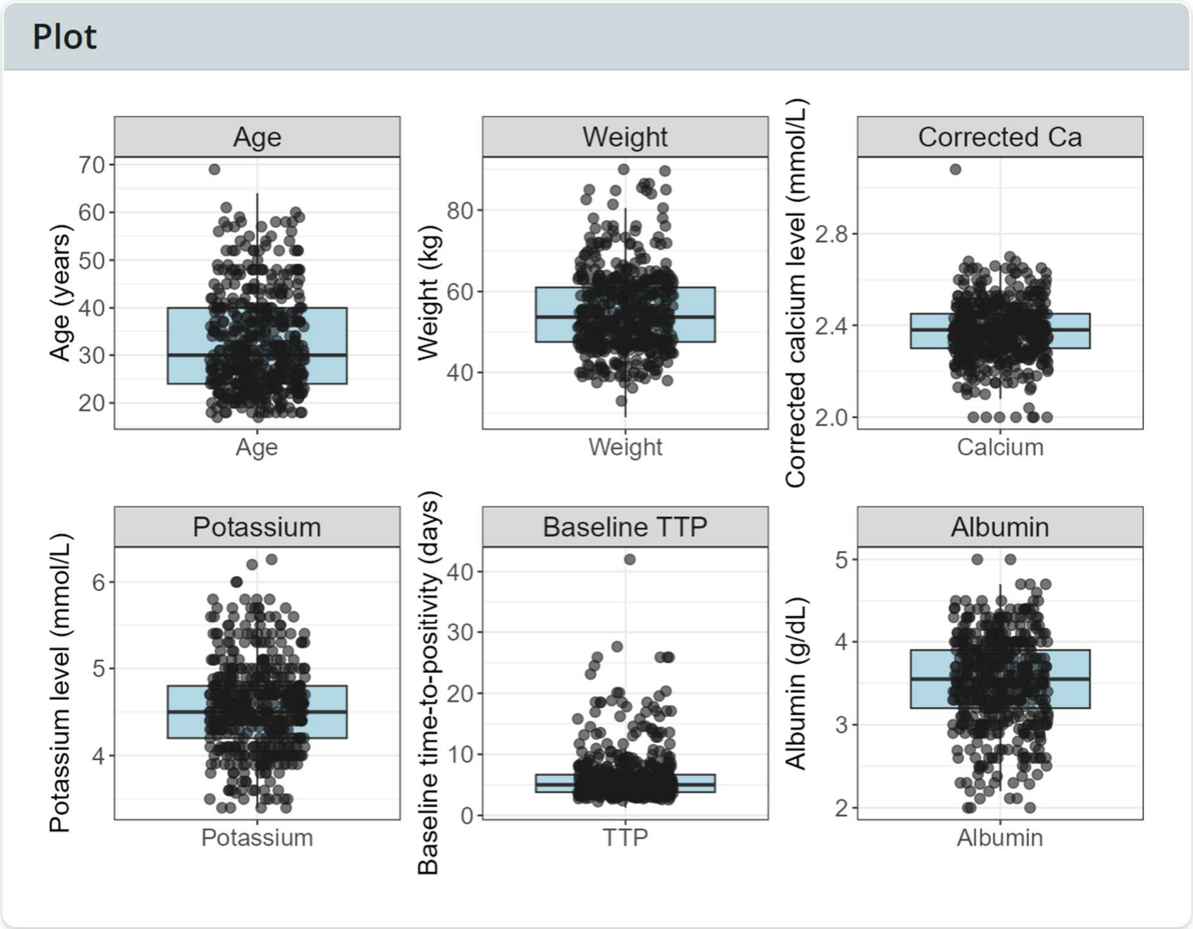

Figure S3. Screenshot of the summary plots of baseline continuous covariates for the sampled 500 patients compared to the large virtual population. The boxplots represent the covariate distribution in the large virtual population using conditional distribution modelling. The dots represent the sampled 500 patients in case study 1 on bedaquiline dose evaluation. Corrected calcium level is calculated using the following equation: corrected calcium level (mmol/L) = measured calcium level (mmol/L) + 0.8 × (4 − albumin level (g/dL)). Ca, calcium; TTP, time-to-positivity.

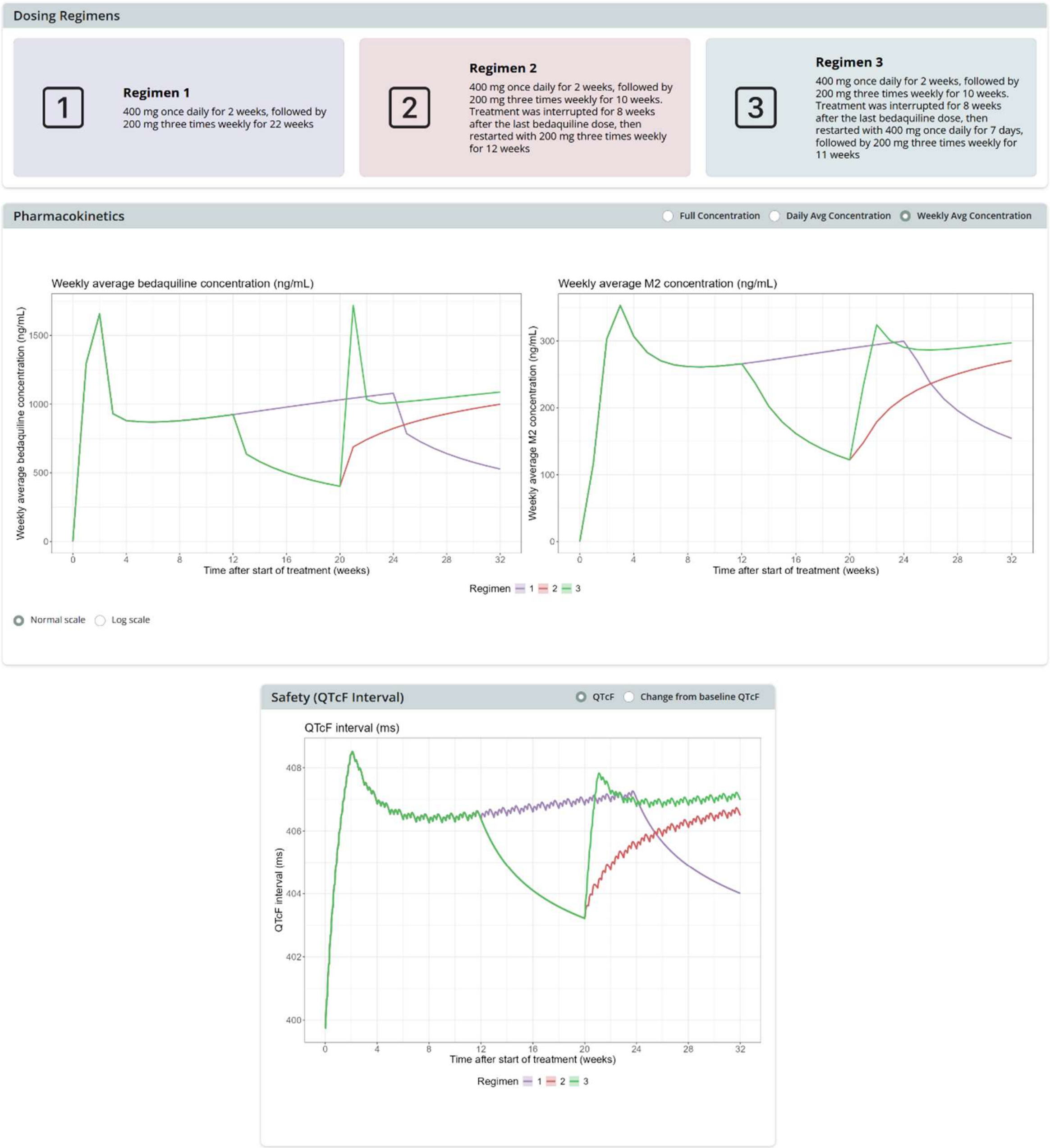

Figure S4. Evaluation of reloading strategies with interruption under the regulatory-approved thrice-weekly dosing regimen for bedaquiline in the Shiny interface app. Regimen 1 (purple): bedaquiline treatment for 24 weeks without interruption. Regimen 2 (red): 8 weeks of interruption after 12 weeks of bedaquiline therapy, and then reinitiated bedaquiline treatment without reloading doses. Regimen 3 (green): 8 weeks of interruption after 12 weeks of bedaquiline therapy, and then reinitiated bedaquiline therapy with a 400 mg daily dose for 7 days. Simulations were performed for a typical individual (32-year-old non-black male weighing 56.6 kg, with baseline albumin 3.65 g/dL, albumin-corrected calcium 2.44 mmol/L, potassium 4.2 mmol/L).

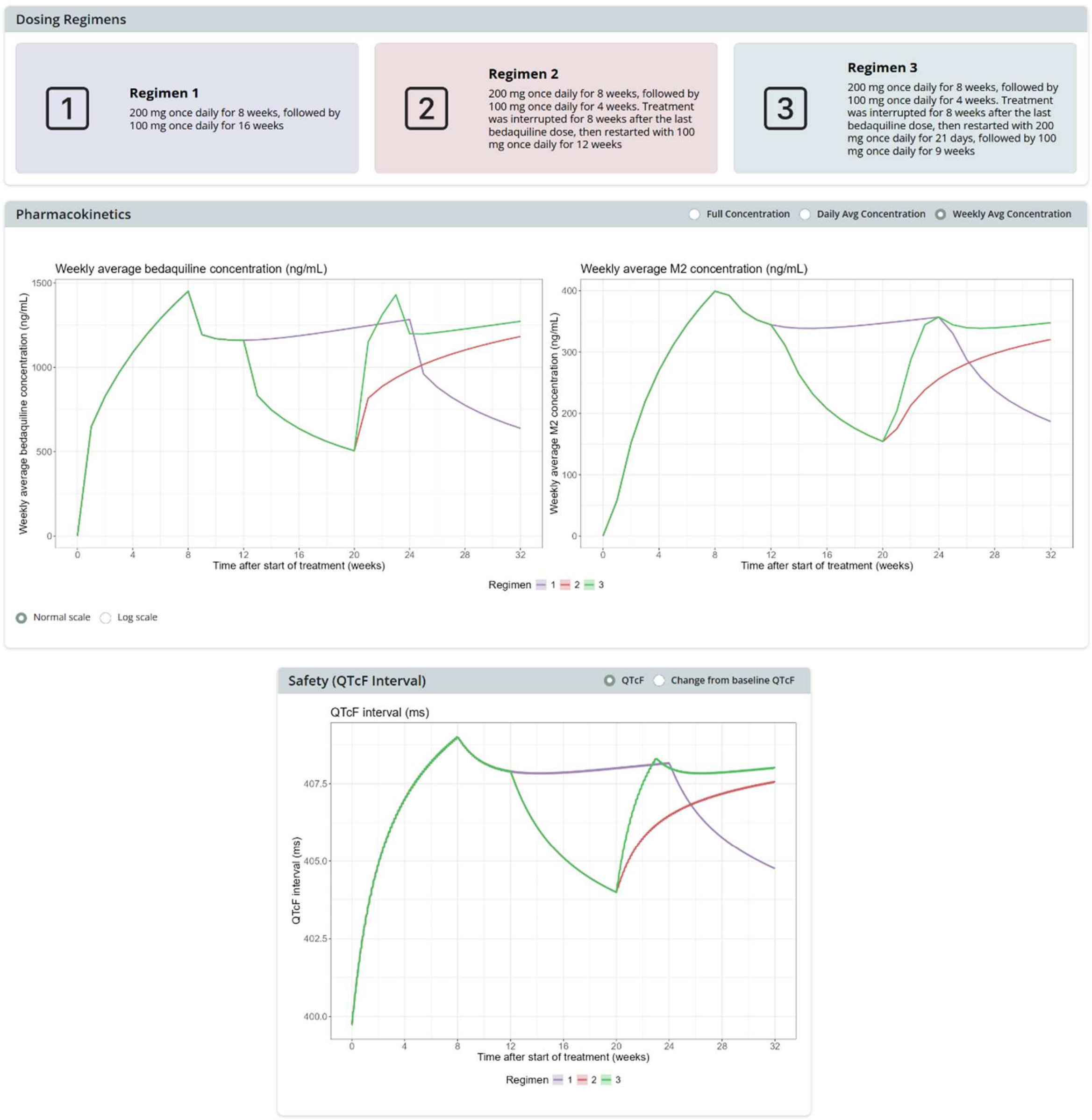

Figure S5. Evaluation of reloading strategies with interruption under the ZeNix once-daily dosing regimen for bedaquiline in the Shiny interface app. Regimen 1 (purple): bedaquiline treatment for 24 weeks without interruption. Regimen 2 (red): 8 weeks of interruption after 12 weeks of bedaquiline therapy, and then reinitiated bedaquiline treatment without reloading doses. Regimen 3 (green): 8 weeks of interruption after 12 weeks of bedaquiline therapy, and then reinitiated bedaquiline therapy with a 200 mg daily dose for 21 days. Simulations were performed for a typical individual (32-year-old non-black male weighing 56.6 kg, with baseline albumin 3.65 g/dL, albumin-corrected calcium 2.44 mmol/L, potassium 4.2 mmol/L).

Additional feature in the Shiny application: Simulation of time to positivity (TTP) data

The Shiny platform supports simulation of individual TTP data using the PK-efficacy model developed by Svensson and Karlsson (*J Antimicrob Chemother*, 2017) under the "Extra: TTP Simulation" tab. Users need to specify the number of individuals to simulate, baseline TTP distribution in a population, half-life of mycobacterial decline (reflecting how different resistance levels and background regimens influence bacterial elimination), sampling time points for cultures, number of culture replicates, individual TTP profiles and definition of time to sputum culture conversion. This function is not limited to bedaquiline-containing regimens but can be used in any other anti-tuberculosis combination therapy, provided the appropriate half-life parameter is specified. This allows users to utilize the outputs of individual TTP data for further analysis, e.g., power calculation. Figure S6 displays the interface of the simulation TTP tool.

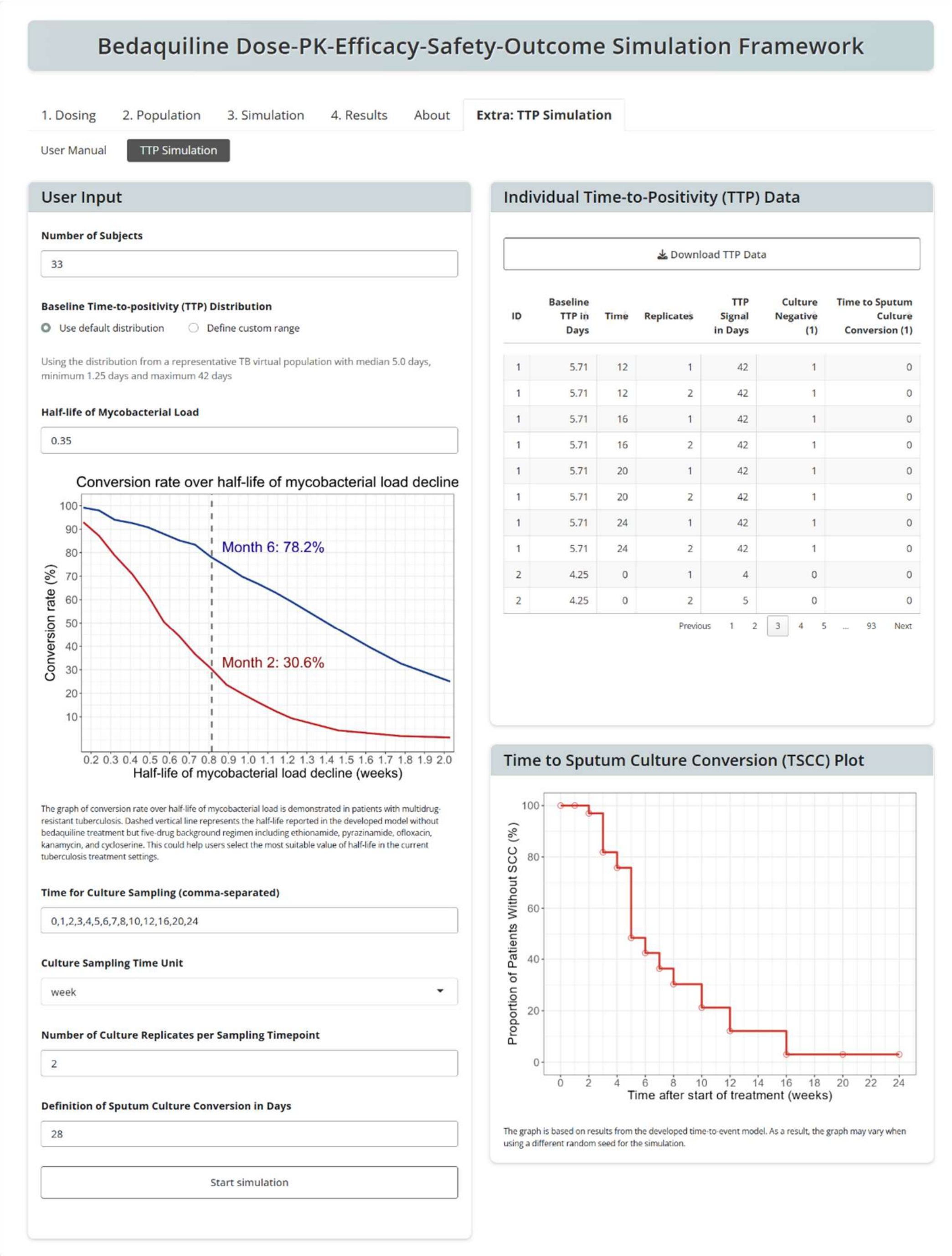

Figure S6. Screenshot of the Shiny interface to simulate individual time-to-positivity (TTP) data. The simulation tool allows users to define simulation settings, including baseline TTP distribution, half-life of mycobacterial load decline, culture sampling schedule, number of culture replicates, and definition of sputum culture conversion (left panel). The simulation outputs include individual-level TTP data (top right) and a time to sputum culture conversion plot (bottom right, only applicable when culture sampling time unit is in weeks). A CSV file of the simulation dataset together with a data specification text file can be downloaded by clicking "Download TTP data".
